# Parents’ Perspectives on the Utility of Genomic Sequencing in the Neonatal Intensive Care Unit

**DOI:** 10.3390/jpm13071026

**Published:** 2023-06-21

**Authors:** Amy A. Lemke, Michelle L. Thompson, Emily C. Gimpel, Katelyn C. McNamara, Carla A. Rich, Candice R. Finnila, Meagan E. Cochran, James M. J. Lawlor, Kelly M. East, Kevin M. Bowling, Donald R. Latner, Susan M. Hiatt, Michelle D. Amaral, Whitley V. Kelley, Veronica Greve, David E. Gray, Stephanie A. Felker, Hannah Meddaugh, Ashley Cannon, Amanda Luedecke, Kelly E. Jackson, Laura G. Hendon, Hillary M. Janani, Marla Johnston, Lee Ann Merin, Sarah L. Deans, Carly Tuura, Trent Hughes, Heather Williams, Kelly Laborde, Matthew B. Neu, Jessica Patrick-Esteve, Anna C. E. Hurst, Brian M. Kirmse, Renate Savich, Steven B. Spedale, Sara J. Knight, Gregory S. Barsh, Bruce R. Korf, Gregory M. Cooper, Kyle B. Brothers

**Affiliations:** 1 Department of Pediatrics, Norton Children’s Research Institute, University of Louisville School of Medicine, Louisville, KY 40202, USAkyle.brothers@louisville.edu (K.B.B.); 2 HudsonAlpha Institute for Biotechnology, Huntsville, AL 35806, USA; 3 Department of Pathology and Immunology, Washington University School of Medicine, St. Louis, MO 63110, USA; 4 Department of Biological Sciences, University of Alabama in Huntsville, Huntsville, AL 35899, USA; 5 Department of Genetics, Ochsner Health System, New Orleans, LA 70121, USA; 6 Department of Genetics, University of Alabama at Birmingham, Birmingham, AL 35294, USA; 7 Division of Genetics, Norton Children’s Genetics Center, University of Louisville School of Medicine, Louisville, KY 40202, USA; 8 Department of Pediatrics, University of Mississippi Medical Center, Jackson, MS 39216, USA; 9 Neonatal Intensive Care Unit, Woman’s Hospital, Baton Rouge, LA 70817, USA; 10 Department of Pediatrics, Children’s Hospital New Orleans, New Orleans, LA 70118, USA; marla.johnston@lcmchealth.org (M.J.);; 11 Department of Pediatrics, University of Alabama at Birmingham, Birmingham, AL 35294, USA; 12 Pediatrics Neonatology Division, University of New Mexico Health Sciences Center, Albuquerque, NM 87106, USA; 13 Department of Internal Medicine, University of Utah, Salt Lake City, UT 84112, USA

**Keywords:** genome sequencing, parent–infant bonding, timing of disclosure of results, parental guilt, utility

## Abstract

Background: It is critical to understand the wide-ranging clinical and non-clinical effects of genome sequencing (GS) for parents in the NICU context. We assessed parents’ experiences with GS as a first-line diagnostic tool for infants with suspected genetic conditions in the NICU. Methods: Parents of newborns (N = 62) suspected of having a genetic condition were recruited across five hospitals in the southeast United States as part of the SouthSeq study. Semi-structured interviews (N = 78) were conducted after parents received their child’s sequencing result (positive, negative, or variants of unknown significance). Thematic analysis was performed on all interviews. Results: Key themes included that (1) GS in infancy is important for reproductive decision making, preparing for the child’s future care, ending the diagnostic odyssey, and sharing results with care providers; (2) the timing of disclosure was acceptable for most parents, although many reported the NICU environment was overwhelming; and (3) parents deny that receiving GS results during infancy exacerbated parent–infant bonding, and reported variable impact on their feelings of guilt. Conclusion: Parents reported that GS during the neonatal period was useful because it provided a “backbone” for their child’s care. Parents did not consistently endorse negative impacts like interference with parent–infant bonding.

## 1. Introduction

Advances in genome sequencing (GS) offer promise in disease risk identification and optimized medical management for critically ill infants. Understanding genomic causes of newborn conditions can facilitate individualized care plans, provide recurrence risk for reproductive planning, and improve patient outcomes [1,2,3]. In newborn intensive care units (NICUs) in the U.S., it is estimated that approximately 10–25% of critically ill infants may have an undiagnosed monogenic condition, thus presenting a missed opportunity for tailored interventions and care pathways [4,5]. In a number of health systems, GS and rapid GS (rGS) is emerging as a first-line test for infants with complex medical conditions to address this issue [6,7,8]. 

Genetic information can also have major non-clinical implications. A person’s evaluation of the personal value of these implications is often referred to as “personal utility”, and may include therapy, schooling interventions, qualification for services, etc. [9]. Recent work has helped inform the development of frameworks for understanding the various ways receipt of a genetic diagnosis might be valuable in the life of a patient and their family. A scoping review of personal utility measures designed to elicit the views of parents with children undergoing genetic testing identified key concepts that included affective, cognitive, behavioral, social, and medical management outcomes [10]. A systematic review of 21 studies identified types of utility that were measured when critically ill infants were undergoing genetic testing, including treatment change, redirection of care, screening or referral, prognostic information, and reproductive information [11]. These and other studies have helped map the categories of utility that might be realized by families [12,13,14]. However, work remains to better understand how the timing of sequencing during infancy might modify various domains of utility: Is there utility that can only be realized if sequencing is performed during infancy? Is there disutility that is created by performing sequencing too early?

The perspectives of parents are key to understanding the value that GS carries for families in the NICU and to anticipate challenges that they might experience. Perhaps most importantly, as clinical practices begin routine use of sequencing in the NICU, it will be important to discern how practical decisions, including testing timeline and pre-test and post-test counseling content, will impact families. We explored a number of these issues by conducting qualitative interviews with the parents of infants who had received GS in NICUs across the southeast United States. We sought to understand how the outcomes involving utilities and disutilities might differ from sequencing performed later in childhood, and how a molecular diagnosis fits into the broader story for families.

## 2. Materials and Methods

### 2.1. Project Overview

SouthSeq, a member of the Clinical Sequencing Evidence-Generating Research (CSER) Consortium, was a mixed-methods translational study to assess the utility of GS in newborns suspected to have a genetic condition based on phenotype. The study enrolled over 600 families and involved GS followed by parent quantitative surveys addressing a variety of outcomes, including a non-inferiority clinical trial comparing disclosure of findings to parents/guardians by genetics providers vs. non-genetics providers (NCT03842995) [15]. The return of results involved standard of care (e.g., return by genetics providers) vs. genetic counselor-trained non-genetics providers (e.g., neonatologist, nurse practitioner) [16]. Providers were not required to use a particular theoretical model of genetic counseling. However, both provider types were provided with patient result letters to guide in result return. Following results disclosure, participants returned to a clinical care pipeline including follow-up with subspecialists. Sequencing outcomes have been published elsewhere [17], and clinical trial results will be published in a subsequent publication. The study was approved by the University of Alabama Institutional Review Board (IRB), and all participants provided written informed consent. We report on findings from the qualitative interview component of this study that was guided by quality standards for qualitative research and the standards for reporting qualitative research [18,19].

### 2.2. Sample and Participant Recruitment

Families were eligible to participate in SouthSeq if their infant (up to 12 months old) was receiving inpatient care and either demonstrated a pattern of congenital variations potentially consistent with an undiagnosed genetic condition, or a major medical condition such as seizures, hypotonia, metabolic abnormalities, etc. [17], without a known cause. For the interview study, purposive sampling was used to select 62 families representing a diverse set of perspectives within our target population. We sought to overrepresent racial and ethnic groups that are underrepresented in genomics research. We did not utilize saturation as a criterion for concluding data collection and instead set the number of research participants a priori. We included families that received a positive result (pathogenic or likely pathogenic), an uncertain result, or a negative result in roughly equal numbers. In a few cases, families were purposively sought for interviews because their experiences highlighted challenges encountered in undergoing sequencing.

One interview was conducted in-person prior to the COVID-19 pandemic, with all remaining interviews by telephone. If more than one parent/guardian per family participated, the interview was separate to ensure each person’s perspectives could be heard clearly. Interviews were audio-recorded, then transcribed by a professional transcription service.

### 2.3. Data Collection

The interview guide was designed to elicit parents’ experience. Domains included the families’ decision to participate in the study, experiences for waiting and receiving the findings, and the personal utility and meaning of primary and secondary findings (Supplementary Materials). Interviews were conducted by study personnel with experience and training conducting semi-structured interviews. We conducted interviews until saturation of key concepts, or where no new insights were emerging, were noted. This is typically reported with a range of 15 to 25 interviews [20,21].

### 2.4. Data Analysis

Qualitative analysis was conducted using the software tool Dedoose (SocioCultural Research Consultants, Los Angeles, CA, USA). Qualitative coding was characterized by a combination of deductive and inductive approaches and proceeded in two rounds [22,23,24]. The initial deductive coding framework was based on an empirically informed conceptual model of perceived utility of genomic sequencing [25], along with several other topics included in the interview guide. Additional codes that fit within this broader framework were added inductively through an iterative process of initial coding, team discussion on proposed codes, and then a further round of coding on the same transcripts to ensure the revised coding scheme was applied uniformly. Every transcript was then coded by one member of the team, and reviewed for accuracy and completeness by a second member of the team. All inconsistencies were then discussed by the entire team to clarify the coding scheme and resolve outstanding inconsistencies. Because coders did not conduct independent coding, intercoder reliability was not assessed [26]. The team then worked iteratively to summarize findings and identify key themes through discussion and independent review of the data, resulting in several summaries of the data.

## 3. Results

### 3.1. Participant Characteristics

Of the 638 infants who underwent GS, 62 families participated in interviews. As a number of parents chose separate interviews, a total of 78 interviews were conducted: 57 mothers and 21 fathers. Approximately half (56%) self-identified as White and one third (33%) as Black/African American. Educational levels ranged from no high school diploma/GED to professional degree (Table 1). Genomic knowledge was also assessed utilizing a validated 15-item tool [27] and the median score was 73.3% (range 53.3–100%) of correctly answered questions.

Genomic results of the 62 probands revealed 19 (31%) with a positive finding, 21 (34%) with an uncertain finding—with two children each having uncertain findings in two different genes—and 22 (35%) with no diagnostic finding (Table 2 and Table 3). Twelve of the uncertain findings were in genes not associated with any condition. The number of days to receive a result after enrollment ranged between 9 and 164 days (median of 85 days). The majority of results (79%) were received when the child was between 31–120 days of age (Table 2).

### 3.2. Interview Findings

#### 3.2.1. Value of Genome Sequencing Result for Child and Family

Participants were asked a series of open-ended questions regarding the impact of their child’s GS results on them, their child, or other family members. One question queried the value of results on reproductive decision making. A mother (negative result) relayed her thoughts about a future pregnancy: “I don’t want to have another baby and this baby has the same problems and we have the same situation and there’s nothing—they don’t know what this diagnosis is or anything. It’s [the GS results] very important”. Another mother (negative result) indicated the result spurred them to consider having more children: “My husband and I agreed that if the genetic testing came back that it is a gene that we passed on to her, we weren’t going to have any more children so we wouldn’t have to go through the same things again. It sounds selfish, but there is really no other way to put it. But at this point, we’ve come to the agreement that we really want one more at least”. One mother (positive result) shared: “[Child] received the recessed gene(s), which is why he ultimately was not compatible with life. It makes us greatly consider our options and our thoughts about trying to have other biological children, which has been one of the most difficult things to come to terms with and grasp. At this time, biological children are off the table because of the results that we received”.

Some participants shared that GS results would help them prepare for their child’s future and care. One mother (uncertain result) explained: “My baby is going to be like this forever. So I guess what I need to do, what I learned that I have to do, is just be there for him, for every time he needs me. He’s going to be like this”. Another mother (negative result) discussed how she wished there could be more information provided so that she could understand what was ahead for her child’s future. She mentioned: “If there was a specific diagnosis just to be able to predict what [Child’s] future would be, just the fear of the unknown. I don’t know the extent of her impairment and what she’ll have to face or go through”.

Many participants described GS results as valuable because it allowed them to know the cause of their child’s condition and end their diagnostic odyssey. One mother (positive result) who had used IVF with both donor sperm and egg discussed her feelings about how the result had brought closure and influenced her decision making: “My plan was once I had my first daughter, that I would wait a year and then I would try again for a second daughter because I wanted two. And so, I needed to know [the GS results]: one for closure for [Child], and then two to make sure that that didn’t happen with my other embryo”. A father (negative result) expressed relief after learning the GS results: “It [GS results] did provide some comfort knowing that both of these things (child’s medical conditions) aren’t a genetic issue and that we shouldn’t see them in future children”.

Participants also described how having a positive GS result helped their child receive focused care by non-physician professions. One father described his experience with a physical therapist: “That’s actually a big thing [having a result and etiology] for us. We’re really focused on that, because a lot of times the therapists focus on his cleft palate. And we actually have a handout for any therapist now, and how that [GS diagnosis] impacts each part of his development”.

Parents discussed benefits related to medical care. A mother (positive result) reported that “It helped us find the medicine to actually help her calm down her seizures”. A father similarly described how this helped organize and coordinate his son’s medical care: “I am so relieved. I couldn’t imagine not knowing [the positive test result]. I couldn’t imagine what the last year of our lives would have been like… Everything we’ve done for him in the last year, not having that as our backbone of where we start in his medical and therapy care”.

#### 3.2.2. Impact on Timing of Results Disclosure

Most results were disclosed when the child was from one to four months of age (Table 2). Participants were asked how they felt about the timing of when they received their results (too early, just about right, too late) and the majority agreed that disclosure was “just about right”. One father (positive result) reported that he felt fortunate they received results in infancy: “Most don’t find out after years and years and years of struggling, trying to figure out the why, and finally having to pay the certain amount of money that an insurance won’t pay for, to get the genetic testing to finally find out why. And we were lucky to find out at three months old, and then tailor his intervention around that”. One mother (negative GS, ~3 months of age) indicated the timing was “perfect to find out about everything” and “because we had known about his condition already for several months before he was born. So we were kind of over the initial, I guess, shock process. And so we were to the point where we were ready to know all that we could”. Conversely, one parent reported that she wished she had learned the results (positive GS, 3 months of age) prenatally because she was dealing with an abnormal ultrasound finding and it could have saved them time researching for answers and from the stress associated with not having a diagnosis.

A number of participants indicated that they did not mind receiving GS results a bit later as it gave them time to process and retain the information. One mother (positive GS, 4.5 months of age) described: “So earlier on in her life we were going through the surgeries and we were going through healing her head and she had to get her chest reopened and you’re not really worried about what caused it; you just want to get it better. And so, when we got the results she had been home for about two months and it was just like a positive thing. Like, okay. This is what it is. We can share this with her when she gets older”. Another mother (uncertain result) similarly indicated that having some time provided an opportunity for research: “We received it [results, 1 month of age] at the right time. We received it where she was stable. She was about to go home, out of the hospital and everything. And then we received the testing results. And that gave us a little more time to look into [whether the result meant their child would have more problems or not] before they even sent her home”.

A few of the participants vacillated when trying to determine if the timing of the results disclosure was right for them. One father (positive GS, 4.5 months of age) explained: “I want to say [results were returned] kind of too early. And I want to say right on time, too, at the same—I want to at least wait until she at least turns one. And once I thought about it though, I said everything happened right on time. It’s best to get it out of the way now while she’s little”. Another father (uncertain, 4 months of age) replied: “I don’t think it was too early or too late. And I can’t really say it was at the right time, because I wouldn’t know what would be the right time for me to receive that”. One mother expressed similar uncertainty about timing of results (negative GS, 2.5 months of age): “Well, I don’t know. That’s a hard question. She had so much going on at that time, that it wasn’t a really good time for us to receive the results. Because I wasn’t, like, in the right headspace at that time to be—to process it, I guess”.

Furthermore, several indicated their focus was on the immediate needs of the child at that time and less on the GS results. One mother (negative GS, 1.5 months of age) shared: “Honestly, it wasn’t at the forefront of our minds, just because of how much he was going through. Every day while we were in the hospital was kind of a fight to keep him alive. So that was kind of our big thing that we were constantly thinking of. And it wasn’t until they started talking about the possibility of him not making it, that we started to wonder about genetic stuff”. One mother (negative GS, 2.5 months of age) reported: “She went through, like, seven or so surgeries… So honestly, I was so preoccupied, I really didn’t have time to think about possibly getting the results. I really had forgotten about the [results disclosure] appointment, when they had called me to let me know that it was on for the next day”.

#### 3.2.3. Effect on Parent–Infant Bonding

In order to explore possible challenges in the use of GS in the NICU setting, we asked participants how they felt about their child’s condition after birth, how it impacted bonding and if GS results influenced them in any way. Many parents described their challenges in bonding, but denied that this was due to their child’s GS result. One mother (negative GS) reported: “We never second-guessed the idea of him not being our child, or not being the child we wanted. But being in the hospital for two months straight, and not getting to hold him for a month, impacted our bond more than his… actual diagnosis”. Many parents described the overwhelming NICU environment and how that interfered with their infant bonding. One mother (negative GS) stated: “So there’s a lot of restrictions just in even holding her. I can’t even move around freely in my arms. It’s not anything that you would do normally with your baby, that bonding. Not being able to feed her. She’s never ate by mouth”.

Another mother (negative GS) described her own distancing after finding out about her child’s critical condition: “I could feel myself trying to pull away, I guess. It’s difficult to describe. There was almost the feeling of revulsion associated very briefly with it”. Another mother mentioned how she had trouble bonding once she learned her baby might not live: “And then, as soon as I found out that she might not live, I just felt like I had to—I couldn’t bond with her… I just had to tell myself, like, this is ‘A’ baby that’s going to die. Like this is not my baby…I didn’t really get to hold her that long after birth. And so the bonding took longer”. Another mother experienced jealousy of the NICU staff because she thought the baby might be bonding more with the staff. She shared: “I wondered if she bonds with other nurses and things more than me…I know that sounds harsh. I don’t know that that is the right worry, I don’t feel like she’s my child. I mean, I do feel like she’s my child. I just don’t feel like I am the parent or the one in control”.

#### 3.2.4. Experience of Parental Guilt

Previous work has indicated parental guilt is another potential challenge in the use of GS during the infant period and nearly all participants described some level of guilt after receiving their child’s GS results.

For some participants, GS results appeared to help alleviate their guilt. One mother felt that her child’s uncertain result explained their medical condition, and when asked whether she experienced guilt she responded: “No, actually no, because it’s genetic. So it’s not anything I could have done. So it was meant to be…honestly, there’s no guilt, no. You just have to make the best out of it”. Other parents described how the results exacerbated guilt. One mother (positive GS result) reported: “I can’t really stay with him, not look at him… it just feels bad, because you find out what happened to him, because of you”. Another mother (uncertain finding) described: “I felt guilty, knowing that I gave it to my daughter, and wondering if it was related to her heart condition. But, as for myself, like it didn’t bother me knowing I have it. It bothered me knowing I gave it to my daughter”. One father (uncertain result) relayed: “I think there’s a little guilt. There’s some toward my wife, too, knowing if she was with someone else, more than likely she wouldn’t have this problem with, ‘am I going to be able to have more kids or not’. So there’s definitely some guilt”. One participant described how she thought her husband felt about passing down the gene identified through GS: “I think it’s just, it’s natural of a parent to blame yourself and feel like it was your fault. And then, when he found out that his dad had one [pathogenic gene variant], he definitely felt guilty. He felt like he passed this on to [our child]”.

## 4. Discussion

Previous work has identified a number of ways GS in the NICU can be valuable to clinicians, including its ability to provide prognostic information, inform tailored care management and referral, improve clinical outcomes, and reduce costs [2,3,28]. Our findings demonstrate that parents value GS for some of the same reasons, especially its potential to inform future care for their child. Ending their diagnostic odyssey helps reduce uncertainty, but also helps them work with providers in and out of healthcare systems to ensure their child is on the most appropriate pathway. One parent suggested that a molecular diagnosis provides a “backbone” for their child’s care, a metaphor that captures the perspectives of many of the parents interviewed. This framing is critical, as it makes it clear why performing GS during infancy, rather than taking a stepwise approach to genetic testing over months or years, can be important to families both in the NICU and after discharge.

Although many parents talked about being overwhelmed while their child received care in the NICU, many nonetheless reported that reproductive decision making was a very present issue for them. Even as one child received care in the NICU, they were considering in vitro fertilization, how to manage a current pregnancy, and whether or not to have future children. Our results thus suggested that delaying GS until later in childhood, or even later in infancy, is likely to be too late when it comes to informing reproductive decision making, not only because parents might become pregnant with another child during this period, but also because for many the uncertainty and concern about future reproductive decision making has already begun.

Perhaps not surprisingly, most study participants reported being satisfied with the timing of GS results return; for most, this took place between 30 and 120 days of age. Our study did not utilize rapid or ultrarapid GS for most participants, however, and others have reported significant interest in getting a diagnosis earlier in critically ill infants for clinical purposes [29]. Many of our parents reported that having some time before receiving genetic results allowed them an opportunity to process the information separate from their situation in the NICU where they were focused on the child’s immediate needs. However, we do not know what parents would have reported if results were disclosed earlier in the child’s course of care. Only six of the sixty-two infants whose parents we interviewed received a genetic result by two months of age, and none prior to one month of age. Future research is needed to directly compare the experiences of parents who receive results very early in their child’s NICU course and those who receive results after the acute phase of care has passed.

Our findings replicate existing literature demonstrating that parents struggle to bond with their sick child while in the NICU. Some have suggested that early genetic diagnosis could also impact bonding with a newborn [30,31]. However, in our study population of NICU infants—most who were quite ill earlier in their course—parents did not perceive the genetic diagnosis itself as a major factor in bonding. However, these findings do not tell us much about how a genetic diagnosis might affect bonding with ill children who receive a diagnosis very early through rapid or ultrarapid sequencing, or otherwise healthy infants who undergo genetic testing through expanded newborn screening; further empirical research will be needed to clarify these issues.

Parental guilt is a universal experience for parents with an ill infant, confirmed by our study, and suggests that genetic results interact with that experience of guilt in complex and sometimes unpredictable ways. Recognition of this is a critical consideration in disclosure of GS findings, and could inform genetic counseling models adapted for the newborn period.

While a strength of our qualitative study included providing rich detail in parent experiences which could help inform future measure development and care strategies, there were a number of limitations. As purposive sampling was used to identify parents with diverse perspectives and critically ill infants with a variety of genetic findings, our results are not assumed to be representative of all parents with children having GS in the NICU. Additionally, this study may be limited due to participant bias: the likelihood that a certain type of participant, or participants with a particular viewpoint, elected to take part in this study. Our research was conducted with English-speaking families and further studies are needed to include non-English-speaking families in these analyses.

A key strategy for addressing questions about timing would be to conduct a trial in which families are randomized to receive genetic testing results either early in the NICU course (such as using rapid or ultra-rapid sequencing), or later in their course. In addition, studies are needed to better understand how genetic counseling and other care strategies might be used to help parents cope with GS results given that they might interpret diagnoses through the lens of parental guilt, or that they might misunderstand negative results to have ruled out a genetic cause. Parents should be made aware that although GS is a fairly comprehensive test, findings may be missed, clinical implications of certain variants may be unknown at time of analysis, and regions of the genome are poorly annotated or have yet to be clinically validated (e.g., complex structural variants, short and long repeat sequences, GC-rich regions). Genome reanalysis should be encouraged, especially in younger cohorts where patient features have yet to manifest until later in development (e.g., intellectual disability, developmental delays). Finally, some of our participants discussed negative responses to having a sick child: one described this as a “revulsion”, and another spoke of an unintentional desire to distance herself from her infant. It is unclear how often parents might experience these types of phenomena, as many may be reticent to admit to having these types of experiences or thoughts. Further research might examine whether this is a common experience, and whether there are strategies that providers might be able to utilize to help parents cope.

## 5. Conclusions

Our study provides insights into the ways parents value having access to GS during the neonatal period, thus helping to clarify our understanding of personal utility during this period. Although guilt and difficulty bonding were commonly reported experiences, most parents rejected the idea that these challenges were exacerbated by GS results. Although follow-up interviews would be useful to develop a longitudinal understanding of the impact of GS in the NICU and throughout the neonatal period, these findings generally support the use of GS during this time period. In particular, our results do not support delaying genetic testing, including GS, to avoid interfering with parent–infant bonding or overwhelming families early in their journey caring for a child with a complex medical condition. Parents value having this “backbone” early in their journey.

## Figures and Tables

**Table 1:** Characteristics of interviewed adults (N = 78).

Characteristics	N	%
**Relationship to child**		
Biological mother	57	73
Biological father	21	27
**Self-reported race or ethnicity**		
Black or African American	26	33
White	44	56
Other	1	1
Native Hawaiian or Pacific Islander	0	0
Middle Eastern, North African or Mediterranean	1	1
Native American or Alaska Native	2	3
Asian	2	3
Hispanic or Latino	2	3
**Educational level**		
Some high school, no diploma	2	4
High school graduate	8	18
Vocational training, incomplete	8	18
Vocational training, completed	6	13
Associate’s degree	4	9
Bachelor’s degree	10	22
Master’s degree	6	13
Professional degree	1	2
No response	45	58

**Table 2:** Characteristics of children (probands) (N = 62).

Result	N	%
Positive (at least one P/LP result)	19	31
Negative (no P/LP or VUS results)	22	35
Uncertain (at least one VUS result)	21	34
**Days to receipt of results (days)**		
0–15	2	3.2
16–30	0	0
31–60	10	16.1
61–90	25	40.3
91–120	14	22.5
121–199	8	12.9
200–300	0	0
>300	0	0
NA	2	3.2
**Age at disclosure of results (days)**		
0–30	0	0
31–60	6	9.6
61–90	18	29
91–120	10	16.1
121–150	14	22.6
151–180	6	9.7
181–210	1	1.6
211–240	2	3.2
241–270	1	1.6
NA	2	3.2

**Table 3:** OMIM or ORDO condition of each proband.

**Positive Result (MIM, ORPHA)**
Mandibulofacial dysostosis, Guion-Almeida type (610536)
Combined D-2- and L-2-hydroxyglutaric aciduria (615182)
Chromosome 4q deletion syndrome (ORPHA: 262029)
Developmental and epileptic encephalopathy 11 (613721)
Sudden cardiac failure, infantile (617222)
Kaufman oculocerebrofacial syndrome (244450)
Severe achondroplasia with developmental delay and acanthosis nigricans (SADDAN; 616482)
Orofaciodigital syndrome XIV (615948)
Adams-Oliver syndrome 6 (616589)
Mandibulofacial dysostosis, Guion-Almeida type (610536)
Catel-Manzke syndrome (616145)
Adams-Oliver syndrome 5 (616028)
Adrenal insufficiency, congenital, with 46XY sex reversal, partial or complete (613743)
Ectrodactyly, ectodermal dysplasia, and cleft lip/palate syndrome 3 (604292)
Lethal congenital contracture syndrome 7 (616286)
4p16.3 microduplication syndrome (ORPHA: 96072)
Hydrocephalus, congenital, 2, with or without brain or eye anomalies (615219)
CHARGE syndrome (214800)
Intellectual developmental disorder, X-linked syndromic, Nascimento type (300860)
**Uncertain result (MIM, ORPHA)**
Mitochondrial complex V deficiency, nuclear type 1 (604273) ^1^
Pancreatic agenesis and congenital heart defects (600001)
Developmental and epileptic encephalopathy 73 (618379)
Smith-Lemli-Opitz syndrome (270400) ^2^
Myasthenic syndrome, congenital, 22 (616224)
Axenfeld-Rieger syndrome, type 3 (602482)
Glycogen storage disease II (232300) ^1^; Ullrich congenital muscular dystrophy 2 (616470)
Cardiac, facial, and digital anomalies with developmental delay (618164)
Arthrogryposis multiplex congenita 5 (618947)
Cardiomyopathy, hypertrophic, 1 (192600)

^1^ Patients were heterozygous for pathogenic variants associated with the recessive condition listed. A second allele could not be identified but the participant had strong phenotype overlap that warranted return. Overall, these cases were considered uncertain due to insufficient zygosity. ^2^ Patient also received an uncertain result that had no associated condition.

## Data Availability

The authors will make relevant anonymized interview data available to collaborators external to our team on email request, assuming that we can make appropriate arrangements to safeguard any sensitive information participants might have revealed in the course of the interviews. The genomic data are available through AnVIL and dbGAP.

## Supplementary Materials

The following supporting information can be downloaded at: https://www.mdpi.com/article/10.3390/jpm13071026/s1. Interview Guide.
