# Supplementary material for: Parents’ Perspectives on the Utility of Genomic Sequencing in the Neonatal Intensive Care Unit
Source: J Pers Med. 2023 Jun 21;13(7):1026. doi: 10.3390/jpm13071026 (PMC10382030; doi:10.3390/jpm13071026)
Supplement: Supplementary file 1 [file jpm-13-01026-s001.zip › jpm-2432167-supplementary.pdf]

CSER SouthSeq Discussion Guide

Greeting

1. [Diagnosis Experiences]

➢ **First, I would like to talk with you about your experiences in trying to find the cause for NAME's medical condition.**

- When did you first become concerned about NAME?
  - What caused you to be concerned?
  - What did you do about your concerns?
- Can you tell me the story of how you and your family have searched for a cause of NAME's condition?
- What has been the *hardest* part so far in searching for the cause of his/her condition?
- What has been the *best* part so far in searching for the cause of his/her condition?

2. [Interest in and Reasons for a Diagnosis]

➢ **Next I would like to talk a little bit about your interest and reasoning in finding the cause for NAME's condition.**

- Think about how you felt right before you started this study.
  Did you feel it was important to know the cause of NAME's condition?
  - Why was it important (or not important) to know?
  - What were the one or two most important reasons for wanting to know the cause of NAME's condition?
- Were there any reasons that made you want to *avoid* knowing the cause?
  [Probe]
- Had your interest in knowing the cause changed since the time you first learned that NAME had this condition? [Probe]

3. [Expectations]

➢ **Let's talk now about your expectations regarding NAME's genetic testing in this study.**

- What were your thoughts when you first heard about this study?

Next I am going to ask you about your *expectations* for this study and then I will ask about your *hopes* for this study.

- What did you expect to learn from these tests?
  - In what way were your expectations met?
  - In what way were your expectations *not* met?
- What you *expected* to learn from these tests, was that different from what you had *hoped* to learn from the tests?
  - Can you tell me about that? [PROBE for differences between 'hoped for' and 'expected.']

4. [Parental Emotions re Child]

➢ **For some parents, the search for a cause to their child's condition can be emotional.**

Learned Prenatally:

**If parents <u>knew prenatally</u> their child might have a medical problem,**<br>**Ask:**<br>• **During your pregnancy**, how would you describe your emotional state in relation to his/her medical problem?<br>PROBE for emotional states:<br>▪ Positive feelings/states, such as hope, acceptance<br>▪ Negative feelings/states, such as despair, anxiety, denial<br>▪ Conflicting simultaneous positive and negative feelings.<br>• Did NAME'S condition affect how you bonded with him/her?

Learned at Birth:

**If parents <u>learned at birth</u> their child might have a medical problem,**
**If parents <u>learned later</u> their child might have a medical problem,**<br>**Ask:**<br>• **When you learned NAME had a medical problem**, how would you describe your emotional state in relation to that problem?<br>PROBE for emotional states:<br>▪ Positive feelings/states, such as hope, acceptance<br>▪ Negative feelings/states, such as despair, anxiety, denial<br>▪ Conflicting simultaneous positive and negative feelings.<br>• Did NAME'S condition affect how you bonded with him/her?

Learned Later:

- Some families have expressed feeling guilt regarding their child's condition.
  Did you at any time experience a feeling of guilt?
  - **If yes**, can you tell me about those feelings?
- ***While you waited*** for the genetic test results for NAME, what were you feeling?
  PROBE for emotional states:
  - Positive feelings /states such as hope, acceptance
  - Negative feelings such as despair, anxiety, denial
  - Conflicting simultaneous positive and negative feelings
- Tell me about your experience when you received NAME'S test results.
  - Who gave you the genetic results?
  - Where were you when they gave you the results?
- ***When you got the test results*** for NAME, what did you feel?

PROBE for emotional states:
    - Positive feelings/states, such as hope, acceptance
    - Negative feelings/states, such as despair, anxiety, denial
    - Conflicting simultaneous positive and negative feelings.
- Did knowing the genetic results affect / change the way you bonded with NAME?
- Would you say the genetic results from this study for NAME:
  - Caused you emotional distress?
    - [If Yes] In what ways?
    - [If No] Why do you think you felt no distress?
  - Provided emotional relief?
    - [If Yes] In what ways?

      [If No] Why do you think you feel no relief?
- What was good about the way you received NAME'S genetic results?
- What could have been better?
- Would you say you received the genetic results too early in NAME'S life, too late, or at about the right time?

5. [Parents' Results]

➢ **Let's set aside the genetic results for <u>NAME's</u> condition for a minute and talk about any results you may have gotten about yourself.**

- Did you get any genetic results about yourself or for OTHER PARENT?
  - [If yes] Can you tell me what you learned?

    PROBE:
    - Emotions
    - Meaning to person, family, extended family
  - [If yes] Do you/OTHER PARENT have a medical problem that is explained by your/OTHER PARENT'S genetic result?
  - As far as you know, are there any medical conditions in your/OTHER PARENT'S family history that can be explained by your/OTHER PARENT'S genetic result?

6. [Personal Utility/Meaning]

➢**Thinking about the genetic test for your child (<u>*and*</u> for yourself/OTHER PARENT), we want to learn if and how the results impacted your lives and the lives of other family members.**

- Taken as a whole, would you say that you were emotionally better before or after you learned the genetic test results from the study—or did you feel no emotional change? [Probe]
- Did you learn anything about your child's genes, your genes, or his/her OTHER PARENT'S genes that distressed you?
  - [If Yes], Can you tell me more about what you learned?
- Did you learn anything about your child's genes that were important or made a difference for him/her?
  - [If Yes], Probe:
    - Changes in medical care
    - Changes in therapy (speech, physical, etc)

- Have you done anything differently in your life since learning something about all your genes?
    - [If Yes] Probe.
- Did you learn anything about your child's gene, your genes, or OTHER PARENT'S genes that you think matters for other people in your family?
    - If yes, have you told them about this?
    - Do you think this was important or made a difference for you or for them?
        - [If Yes] Probe.

[If applicable]

- As far as you know, have any family members done anything differently since learning something about their genes from this study?
    - [If Yes] Probe.

7. [Reproductive Decisions]

➢ **The next topic I would like to discuss with you is about future pregnancies.**

- Did you learn anything from the genetic test in this study that has changed the way you think about having more children? [Explore]
    - [If Yes] How has your thinking about having more children changed?
    - [If No] Why do you think the test results did ***not*** change how you feel about having more children?
- In thinking about having more children, would you say that you are better off now than you were before you received the genetic test results?

8. [Results and Educational Materials]

➢ **The last topic I would like to discuss with you is about your experience with the written genetic test report you received from the study**

- Were you able to understand the report?
    - Yes: Are there specific things about the report that made it easy to understand?
    - No: Are there specific things about the report that made it difficult to understand?
- Is there any information you wished was on the report that was not there?
- Have you used any of the links and resources that were mentioned on the report?
- Have you shared the report with anyone?
    - Yes: Who have you shared the report with?
    - No: Are you planning to share the report with anyone?

We would also like to ask you about your experience with the Genome Gateway website.

- Did you have difficulty accessing or navigating the Genome Gateway site?
    - Yes: Probe for specific difficulties
        - Logging in/remembering website URL/password
        - Unable to read on particular type of device
        - Finding educational information
        - Finding questionnaires
        - Playing videos
        - Downloading files

        - Messaging study staff
    - No: Is there anything specific that made using Genome Gateway easy?

- At what point(s) during your participation in the study did you use Genome Gateway?
    - Right after enrolled
    - While waiting on results
    - After receiving results
    - Combination of 1+ of above

- Thinking specifically about the educational information in Genome Gateway, did you find the material helpful?
    - Yes: What specific topics do you recall being most helpful
    - No: Are there specific topics that you recall being unhelpful or confusing?
    - No: Are there other topics you wish had been covered, but were not?

Did you use any other tools, websites, resources, or online patient support groups to learn more about your/your child's results?

- Yes: What resources did you use? Did you find them helpful?
- No: Probe for why
    - Wanted information that is not available
    - Satisfied with information already received
    - Overwhelmed and not looking for more information
    - Negative result, no reason to look anything up
